# Phase Solubility Changes during Hydration of Monocalciumaluminate and Calcite—The Influence of Alkali Accumulation

**DOI:** 10.3390/ma13061406

**Published:** 2020-03-19

**Authors:** Tanja Manninger, Daniel Jansen, Jürgen Neubauer, Friedlinde Goetz-Neunhoeffer

**Affiliations:** Department of Mineralogy, GeoZentrum Nordbayern, FAU Erlangen-Nuernberg, 91054 Erlangen, Germany; Juergen.Neubauer@fau.de (J.N.); friedlinde.goetz@fau.de (F.G.-N.)

**Keywords:** hydration kinetics, CA, calcite, modelling, pore water

## Abstract

The reaction of CA (monocalcium aluminate) with calcite was closely monitored with regard to phase development, pore water ion content and heat flow. Calcite acts as filler and reactant, finally leading to thermodynamically stable products after hydration at ambient conditions. For better understanding the mechanism taking place, a CA-cement and a commercial calcite mix were compared to a pure CA and pure calcite mix. Both reaction paths were compared. Thermodynamic modeling with PhreeqC gave insight about factors that can influence the course of the hydration reaction. Alkali ions in pore solution of the CA-cement relocate solubility curves of hydration products. Taking into account as many of the alkaline ions as possible, resulted in the closest representation of the measured phase content, confirming thermodynamic modeling. The high dynamics that develop during reaction could only be addressed if a concentration of alkalis in the pore solution at later points in time was respected, thus leading to a shift of solubility curves over time. This was not observed with the pure CA in absence of alkalis.

## 1. Introduction

This study is in close relation to two previous studies on hydration of calcium aluminate cement (CAC) with addition of calcite and an accelerating Li-salt [1,2] and intends to provide insights into the change in solubility of hydrate phases, due to alkali contents in pore solution during hydration of CA-CaCO_3_ mixes.

Calcium aluminate cement is a special use product employed in castable refractories and dry mix mortars for special applications due to its high aluminum content.

The phase CA (monocalcium aluminate) is the main component of widely used CAC. Nevertheless, CACs generally contain CA_2_ (grossite), C_12_A_7_ (mayenite) and series of other solid solutions of the aforementioned chemical compounds carrying Fe, Na, Mg, Ti and K ions [3]. These additional elements can more or less severely impact the hydration properties of a CAC compared to pure CA. Beside Al especially Na ions were found in pore solutions of CAC [4].

Existing studies show how CAC reacts in the presence of those ions (examinations for Na, Li, K, Mg, Sr, Cl etc. were performed). One effect found is an acceleration of the start of the main reaction [5,6,7]. The influence of those ions can intensify during hydration due to accumulation in the pore water. This accumulation is a result of the high solubility of ions like Na and K [1].

CaCO_3_ (calcite) was used as carbonate source and filler. It leads to the early formation of the thermodynamic stable phase monocarbonate (MC) [8], can mitigate conversion [9,10] at w/c ratios above 0.66 and increases mechanical strength [11,12].

Focusing on the processes in the liquid phase there are calculated thermodynamic data available on the equilibrium surfaces of the system CaO-Al_2_O_3_-CaCO_3_-H_2_O, as well as the influence of the ion Na^+^ on the solubility curves of such a system. The calculation of equilibrium surfaces and their shifts when Na is added, as well as its influence on the phase formation were in focus of Damidot et al. [13].

Subsequently our paper provides a new approach, adding more information by combining computed modeling with measured data. Data of a pure and a technical system (including some impurities) determined from experiments where set in relation to thermodynamic calculations of solubility curves. For our calculations not only Na but also K and S were taken into account.

Some points that are specifically covered and discussed in this paper:

- The pore water ion content of the samples during early hydration and its relation to the respective solubility curves and hence the formation of hydration products is compared to measured crystalline phase content.
- Comparison of a pure CA and calcite mixture to a mix of industrial grade materials is provided (some of the data of the latter was already published in a different context [2]).
- Insights are given into expected effects of impurities during the application of industrial grade high alumina CAC in general.

The obtained knowledge is also contributing to a deepening of our general understanding of CA hydration in the presence of calcite.

## 2. Materials and Methods 

The starting materials were pure synthesized CA as well as a commercial CA-cement (with only CA phase) with two calcite sources, pure synthesized and industrial grade calcite, were examined by means of XRF (X-ray fluorescence), QXRD (quantitative X-ray diffraction) and BET (Brunauer–Emmett–Teller method) (data collection from three independent experiments each). All hydration measurements were done at 23 °C with a w/s value of 1.0 (heat-flow calorimetry, in-situ X-ray diffraction). Pore solution data were obtained by ICP-MS (inductively coupled plasma mass spectrometry). For comparison to QXRD thermodynamic modeling of predicted stable phase contents was performed.

### 2.1. Calcium Carbonates

#### 2.1.1. Commercial Calcite

Due to the paste properties and enhancing reproducibility a commercial calcium carbonate (high specific surface area of 21.7 ± 0.1 m²/g BET) was used for experiments with the CA-cement. Acting as a fine filler, leading to a very reproducible start of the main reaction, it also functions as a carbonate source. CA-cement and calcite ratio was 70/30 (wt%) during experiments. Table 1 shows the chemical analysis by XRF and QXRD data obtained by G-factor method [14] can be found in Table 2. P_2_O_5_ contained in the calcite is a modification residue to achieve higher BET surface. No crystalline phase was detected by XRD but instead amorphous content. 

#### 2.1.2. High Purity Calcite

The pure calcite was used in combination with the synthesized CA in order to avoid introduction of foreign ions. The producer gives a purity of >99.5 % with a sulfate content of ≤0.01 %. The high purity calcite has a BET surface area of 0.8 ± 0.2 m²/g.

XRF analysis data are given in Table 1 and QXRD results obtained by G-factor method can be found in Table 2.

### 2.2. Characterization of the CA-Cement

The commercial CA-cement used contains mainly CA. G-factor method [14] shows a CA_2_ content of 4.7 ± 0.2 wt%. and 8.5 wt% amorphous phase. Chemical composition by XRF is shown in Table 1, Table 2 provides the data of QXRD phase analysis. A value of 2.51 ± 0.05 m²/g for BET surface area was determined (measured with Gemini 2360 Micromeritics, Micromeritics Instrument Corp., Norcross, GA, USA). Initial Na release was tested by shaking the pure CA-cement with deionized H_2_O for ten minutes (w/c = 0.7—similar to used mixtures). A readily soluble Na concentration of 2.3 ±0.1 mmol/L was measured.

### 2.3. Characterization of Pure CA

The material was synthesized in platinum crucibles from a stoichiometric mix of CaCO_3_ and Al_2_O_3_ by milling followed by calcination at 1000 °C, then the mix was re-milled and afterwards sintered at 1400 °C. A BET surface of area of 0.6 ± 0.1 m²/g was measured. A test of Na release (CA + H_2_O w/c = 0.7, shaken for 10 min.) revealed a readily soluble Na concentration ≤0.1 mmol/L.

XRF analysis data was obtained (Table 1) and QXRD results by G-factor method can be found in Table 2.

### 2.4. Sample Composition for Hydration Experiments

The compositions of the CA-cement mixture and the pure mixture are given in Table 3.

### 2.5. Heat Flow Calorimetry

In order to obtain comparable data, isothermal heat flow calorimetry was performed for all mixes at 23.0 °C to determine hydration behaviour. All tests were accomplished with a TAM Air calorimeter (TA Instruments, New Castle, DE, USA). The device was equipped with InMixEr tools (a customized solution for sample equilibration and mixing of water and cement inside a calorimeter [15]). To ensure sufficient equilibration the base line was observed. Before the measurement was started the base line was set to zero. Water injection into the mixture was chosen as starting point for data recording. Homogeneity of the samples was achieved by manual stirring for 30 s and then stirring with an external motor (constant stirring rate of 715 rpm) for 1 min. Three independent measurements were performed. 

### 2.6. Pore Solution Analysis and Description of Thermodynamic Calculations

Pore solution extraction started from 0.25 h until end of main period of hydration for each system examined. Containers of the dry mixes of CA-source plus CaCO_3_-source (each ca. 50 g) and sealed containers containing H_2_O were temperature-equilibrated for 10 h at 23.0 ± 0.1 °C. H_2_O was stirred with the dry powder for 1 min. Pore solution extractions were performed for the mixtures after following intervals considering the heat flow curve (0.25, 0.5, 1, 2.5, 3, 3.5, 4, 5, 6, 8 h for the CA-cement mix and 0.25, 1, 5, 10, 15, 17, 20, 22, 25 h with the pure CA mix) at 23 ± 0.1 °C. Different extraction methods were required due to paste workability. Pastes still showing workability were centrifuged for 8 min (3220 g). For samples after setting, pore solution extraction by pressing (pressure ≤ 47.6 N/mm^2^) was deployed. A hydraulic press (Stürmer Metallkraft wpp30, Stürmer Maschinen, Hallstadt, Germany) equipped with an extraction cell (Remt Industries, Grena, France) was used. Resulting pore solution was filtered (0.2 µm syringe filter) and HNO_3_ was added in defined amounts in order to prevent precipitation. Ion concentrations of Ca, Al, Na, K and S were investigated with a Thermo iCapQ ICP-MS (ThermoFisher, Langenselbold, Germany). To determine inorganic carbon content CO_2_ concentration exetainer vials containing defined amounts of pore solution and HNO_3_ were used. Analyses were carried out on an IRMS Delta plus XP with a Gasbench II (ThermoFisher, Langenselbold, Germany). Blank value for inorganic carbon in deionized H_2_O was <0.05 mmol/L.

The thermodynamic database Cemdata 18 [16] was used in order to calculate the solubility curves in the system Na/K-Ca-Al-C. The advantage of such calculations and plots arises from the possibility to plot the evolution of the pore solution. The diagrams allow to visualize the equilibrium of the pore solution with the precipitated hydrate phases which helps discussing the results gained from pore solution experiments. With the GEM-Selector 3.3 [17,18,19] and the CEMDATA 18 database [19] the activities of different ion species where calculated considering the measured ion concentrations. Activity coefficients where computed using the extended Debye-Hückel equation. This data was used further, to calculate the solubility curves of hydrates (line where the saturation index is zero) within the system Ca-Al. For further insights into the computation of such curves and the thermodynamic background the authors like to refer to recently published articles [20,21,22]. 

The pH was calculated with the program PhreeqC Interactive 3.4.0-12927 [23] from the pore water ion content with the Cemdata 18 database [16].

### 2.7. QXRD and in-Situ QXRD Procedure and Set-up of Thermodynamic Calculations

A Bruker D8 (Bruker Corporation, Billerica, MA, USA) diffractometer operated at 23 ± 0.1 °C XRD was used to for all XRD experiments. Raw sources of CA and CaCO_3_ were analysed by powder XRD (instrument data Table A1-column dry samples). The measurement parameters (angular range up to 70° 2θ, small step size and counting time/step) were chosen to determine the purity of the material in a precise way. In-situ measurements parameters are listed in Table A1–column in-situ XRD/pastes. The goal was an efficient way to determine the quantitative phase development of crystalline phases during a rapid reaction. Therefore step size and counting time/step where chosen in a way to keep the measurement time for each diffractogram short (10 min per diffractogram). The angular range is also short. It starts at 6° 2θ to avoid missing any hydrate phase peaks. The preparation of the pastes was done by stirring equilibrated dry mixes and H_2_O for 1 min. Then they were applied to a temperature-controlled sample holder. Kapton^®^ X-ray film (RESOGOO GmbH & Co. KG, Westerrönfeld, Germany) was stretched above the samples to reduce evaporation of mixing water. Phase development over time was determined by in-situ measurements up to 40 h. Structure data for Rietveld refinement is presented in Table A2. For quantitative phase content G-Factor method [14,24] was applied with exception of C_2_AH_8_ (specification as scale factor). C_2_AH_x_ phases are shown in arbitrary units of their scale factors in the following figures. 

Theoretical equilibrium phase content of used pastes was calculated (thermodynamic modeling via GEMS 3.3 [17,18,19]) using the CEMDATA 18 database [16] and compared to QXRD data.

## 3. Results

### 3.1. Heat Flow Calorimetry

**Figure 1:**
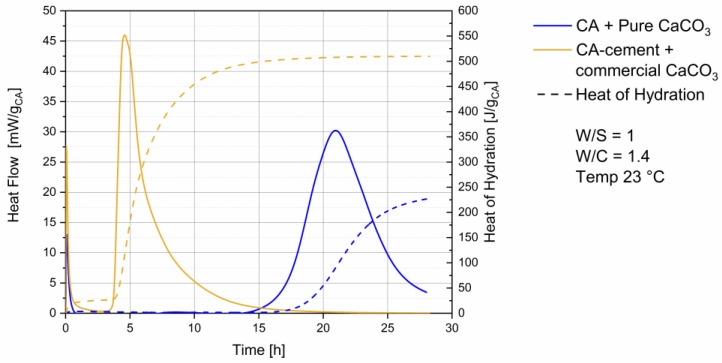
Heat flow & heat of hydration, blue curve CA + pure CaCO_3_, yellow curve CA-cement + commercial CaCO_3_.

Heat flow and heat of hydration of the systems examined are shown in Figure 1. Hydration of the commercial CA cement with a commercial calcite is shown in the yellow curve. A dormant period can be observed after the initial heat flow event. CA with pure CaCO_3_ (blue curve) shows a longer dormant period, with a start of the main reaction at 14 h. The end of the main reaction exceeds 30 h. For the CA-cement (yellow curve) the main reaction starts at 3.5 h and ends after 20 h. At 25 h heat flow reaches again zero.

Particle sizes and surface areas of the reactants are known to have significant influence on the start of cement main reaction, due to the filler effect of the fine [25,26]. In the used samples the different heat flow behavior is mainly a result of the different surface areas and thus both the reactivity of the compounds as well as the filler effect. This is to be expected regarding the use of an industrial product line and a self-synthesized compound. The heat flow was measured to get an overview of the different reaction times, to create a reproducible experimental setting for planning of water extraction. 

Pore solution composition during CAC hydration was found to be dominated by initial CA dissolution [27], which is already happening during mixing in both systems (see first hour in Figure 1) and no influence of particle size distribution can be expected at this time.

### 3.2. Results from Pore Solution Analysis and Thermodynamic Calculations

#### 3.2.1. Pore Solution Data from CA with Pure CaCO_3_

**Figure 2:**
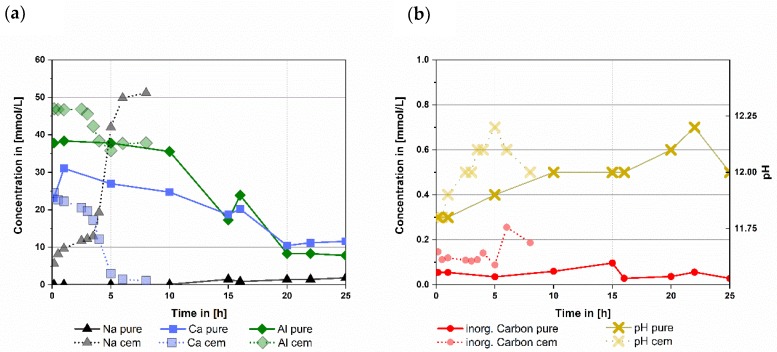
Comparison of ion concentrations (**a**) and inorganic carbon & pH (**b**) of mixtures with pure CA & pure calcite (pure) and the CA-cement & industrial grade calcite (cem) over a time span up to 25 respectively 8 h, (w/s = 1 for both mixtures). Data of the CA-cement & industrial grade calcite were already shown in another context in [2]. ICP-MS error is usually in ng/L range while measured data is in the range of 10 to 600 mg/L, therefore error was not depicted.

For pure CA and CaCO_3_ (Figure 2b) pH values between 11.8 and 12 were calculated during the dormant period (from 0.25 h up to 17 h). During the reaction it peaks at 12.25 at 22 h and declines to 12 at 25 h after the main reaction took place. 

During dormant period Ca content drops from a maximum of 30 mmol/L to 11 mmol/L. Al stays at a concentration of 37 mmol/L until 10 h before it drops to 8 mmol/L. Inorganic carbon is constantly below 0.1 mmol/L during the whole time observed. Na concentration in the pore solution stays at 0.1 mmol/L during the dormant period and increases to 3 mmol/L after the main reaction starts. This is caused by hydration of CA, releasing Na into the pore water (Figure 2a). Additionally, the analysis also shows a sulphur (S) content of 0.9 ± 0.3 mmol/L in the pore water during the whole measurement, which might be a residue from the used calcite.

#### 3.2.2. Pore Solution Data for CA-Cement with Commercial CaCO_3_

For the CA-cement with commercial CaCO_3_ the calculated pH ranged from 11.8 to 12 during the dormant period (Figure 2b from 0.25 h up to 3.5 h). It increases up to 12.25 at 5 h during the main reaction and declines again to 12 at the subsiding of the reaction at 8 h. While the dormant period takes place Ca content declines from 25 mmol/L to 17 mmol/L and Al firstly stays at 47 mmol/L followed by a drop to 42 mmol/L (Figure 2a). Inorganic carbon is constantly between 0.1 and 0.15 mmol/L during the dormant period (Figure 2b). After 3.5 h the main reaction starts. Ca content decreases to 1 mmol/L, Al decreases to 37 mmol/L and inorganic carbon concentration increases to 0.25 mmol/L. Na value starts at 5 mmol/L and increases to 13 mmol/L. After 3.5 h Na increases to 50 mmol/L caused by further hydration of CA and pore water consumption (Figure 2a). 

#### 3.2.3. Comparison of the Pore Solution Evolution in the Systems Examined 

The pure CA with pure CaCO_3_ shows a hydration behavior comparable to the commercial CAC with commercial CaCO_3_ as shown in the chapters before. However, there are some significant differences that will be discussed. 

The highest Ca content measured for the pure CA mix was 31 mmol/L while for CAC it was 24 mmol/L. While the reactions took place the Ca ion contents of pure CA with pure calcite declined ending at 12 mmol/L and for the commercial CAC with commercial calcite 1 mmol/L. 

Aluminum concentrations in the pore solution also dropped during hydration of both systems examined. For CA the starting Al concentration was 38 mmol/L and dropped to 8 mmol/L. In the commercial CAC system Al concentration starts at 47 mmol/L declining to 36 mmol/L at its lowest. 

For the pure CA the Ca to Al ratio at 0.25 h is close to the ratio in dry CA, namely 0.5. This suggests that at the start of the hydration, the Ca and Al ion content is determined by the dissolution of CA. Later the Ca ion content rises.

**Figure 3:**
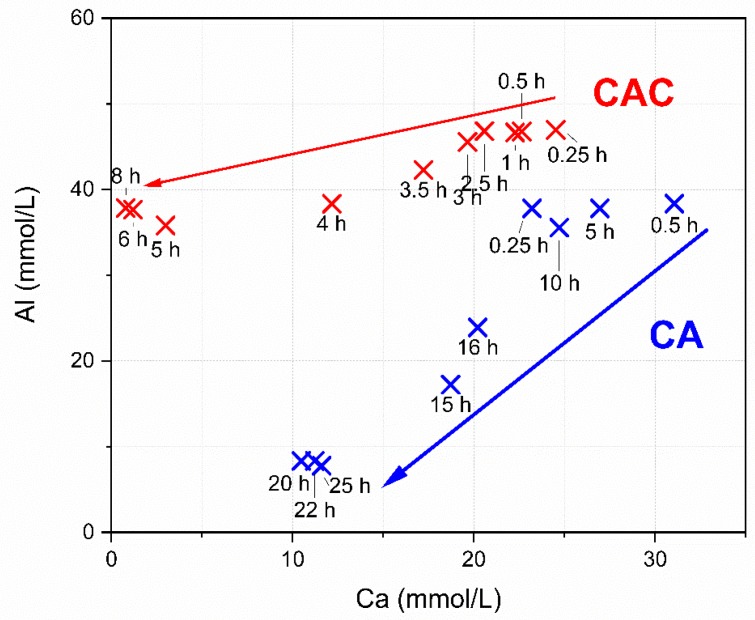
Pore solution evolution of the systems, examined in the solubility system Al-Ca.

The arrows in Figure 3 mark the average path along the Ca and Al ions follow during the reaction (red for the commercial CAC system, blue for the pure CA system). The pure CA mix shows a comparable amount of dissolved Ca ions at early hydration times compared to the CA-cement. During the whole reaction of the pure CA the Al amount dissolved is lower than in the CA-cement. 

In conclusion it can be noted that the pore solution evolves differently in both systems, although it can be seen that the hydration is resulting in the formation of similar hydrate phases. 

### 3.3. Results from in-Situ XRD 

**Figure A1:**
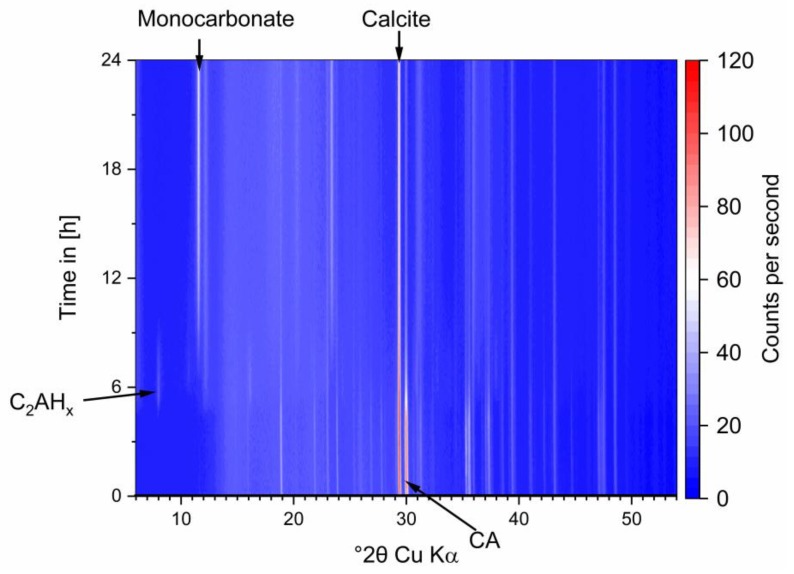
In-situ XRD data of the pure system during hydration.

**Figure A2:**
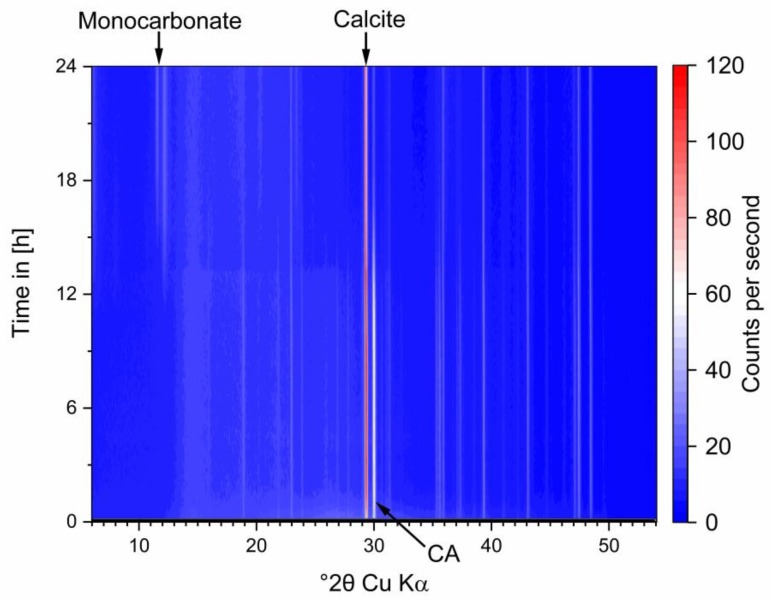
In-situ XRD data of the cement system during hydration.

For XRD level plots see Figure A1 and Figure A2.

#### 3.3.1. Phase Development of Synthetic CA with Pure CaCO_3_

**Figure 4:**
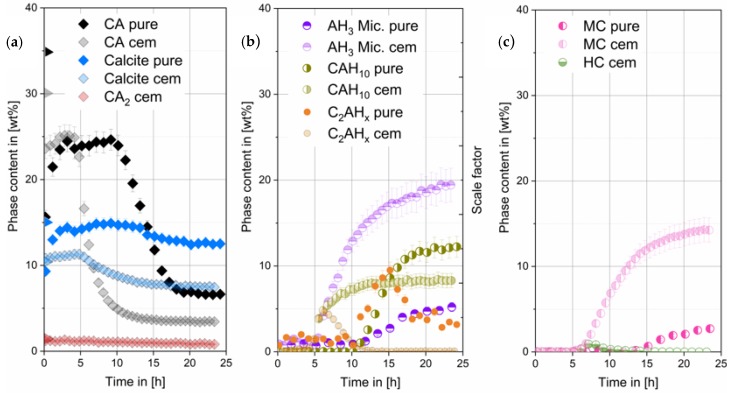
Crystalline phases of the CA & pure calcite (pure) compared to the CA-cement & industrial grade calcite (cem) (obtained by G-factor quantification, w/s = 1). CA quantification error at the beginning due to H_2_O film formation on sample surface (**a**). Scale factors of C_2_AH_x_ phases (arbitrary units) (**b**). In (**c**) MC and HC content in the different samples are compared. Data of the CA-cement & industrial grade calcite were already shown in different context in [2].

Theoretical crystalline CA amount in the mixed paste at hydration start should be 35 wt%. The detected amount of CA in the paste before the main reaction is the result of a thin water film on top of the XRD sample leading to a small underquantification of CA. After 25 h initial amounts have decreased to 7 wt%. During hydration (see Figure 4) C_2_AH_x_ phases were detected from 11 h on, increasing until 15 h and dissolving again until end of measurement. CAH_10_ and AH_3_ formation starts after 12 h. There is a slight deviation to the start of the heat flow at 14 h observed in Figure 1. The difference can be attributed to manual preparation. After 15 h precipitation of monocarbonate (MC) was observed. 

#### 3.3.2. Phase Development of CA-Cement with Commercial CaCO_3_

Initial crystalline CA amount in the mixed paste at hydration start was calculated and should be 30 wt%. A small underquantification can be seen. However, after 15 h initial amounts have decreased to 3 wt%. Phase content of CA_2_ does not change significantly within the 20 h measured in our experiments (see Figure 4a). During hydration (see Figure 4b) C_2_AH_x_ phases were detected from 3.5 h onwards. C_2_AH_x_ amount increases until 6 h followed by a drop to zero after 15 h. CAH_10_ and AH_3_ formation starts slightly before 5 h, correlating well with the observed heat flow in Figure 1. After 5 h precipitation of hemicarbonate (HC) and monocarbonate (MC) was observed. Hemicarbonate content reaches a maximum at 7 h, then decreases reaching zero after 15 h (see Figure 4c). 

## 4. Summary and Discussion

Although both samples mainly contain CaAl_2_O_4_ (CA) as a reactive phase, the CAC shows a number of impurities, especially alkalis (determined by solubility test). Those are a result of the production of the contained Al_2_O_3_ in the cement from the Bayer process and do influence the hydration reaction. 

Especially the solubilities of the hydrate phases formed can be influenced through the presence of alkalis. Additionally, dissolution of the oxides influences the pH of the pore solution of a cement.

### 4.1. Thermodynamic Stable Phases Calculated with GEMS

**Figure 5:**
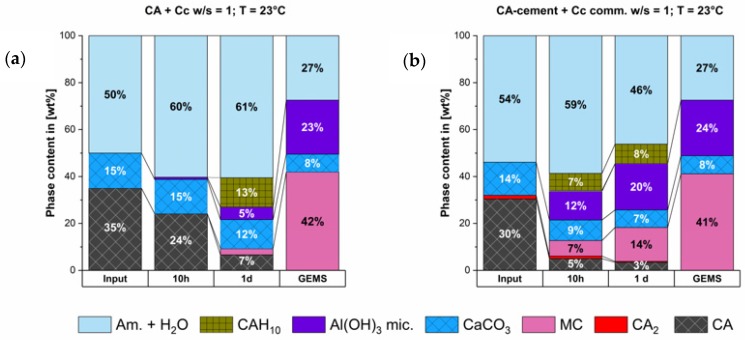
Calculated thermodynamic stable phases compared to measured phase content at 10 h & 1 d of the synthesized CA & calcite (**a**) and the CA-cement & commercial calcite (**b**).

Figure 5 shows the phase development regarding the thermodynamic stable outcome that was predicted by GEMS calculation. The main stable phases that should develop are monocarbonate (MC) and AH_3_. Both phases are formed, as predicted by the thermodynamic model.

As it can be seen from modelling the initially formed C_2_AH_x_ phase is not stable in the system. Hence, the dissolution of this phase as measured is in accordance with the equilibrium in the system. 

CAH_10_ is also formed and is still present at end of our measurement, although it is not thermodynamically stable. However, since our examination duration is only one day it is possible that the destabilization and dissolution of the initially formed CAH_10_ phase is much slower compared to the dissolution of the C_2_AH_x_ phase. Therefore, it does not take place within our examination period. There is a surplus of CaCO_3_ which will not react and hence, acts as a filler. In both systems the predicted phase composition at equilibrium conditions could not be reached within the first day of hydration. 

### 4.2. Solubility Curves and the Pure CA Mix

**Figure 6:**
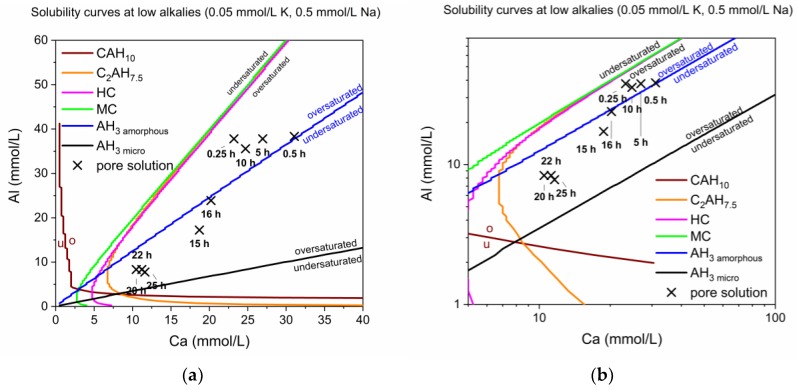
(**a**) Calculated solubility curves at low alkalis (0.05 mmol/L K, 0.5 mmol/L Na) with plotted Ca and Al ion content over time in the pure CA & calcite mix. (**b**) A logarithmic presentation of (**a**), giving insight in the effect of small scale changes at low Ca content, showing solubility curves at low alkalis (0.05 mmol/L K, 0.5 mmol/L Na) with plotted Ca and Al ion content over time in the pure CA & calcite mix.

The first calculations of solubility curves with PhreeqC took into consideration the following ions: Ca, Al, Na, K. The solubility curves for the phases of interest in the system Ca Al at low alkali concentrations (0.05 mmol/L K, 0.5 mmol/L Na) are shown in Figure 6.

The Ca to Al ratio passes different stability fields during hydration (see Figure 6). The solution generally is supersaturated in AH_3_ but passes from possible precipitation of amorphous AH_3_ to the microcrystalline modification. Different specimen of AH_3_ were described and discussed by Lothenbach et al. [28]. Precipitation of AH_3_ with a low crystallinity was observed by QXRD from 12 h onwards. Nevertheless, the calculated solubility curves and the measured ionic concentrations do not fit very well if the XRD-measured phase assemblages are considered. E.g., C_2_AH_x_ is always calculated as supersaturated although a clear decline was observed by XRD in reality.

**Figure 7:**
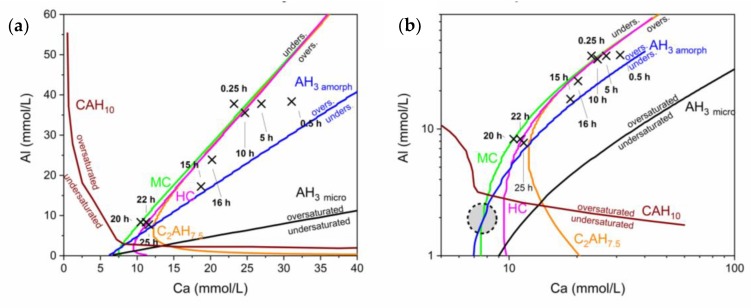
(**a**) Solubility curves at low alkalis (0.05 mmol/L K, 0.5 mmol/L Na) and with the consideration of a minor amount of S in the pore solution (0.9 ± 0.3 mmol/L). The marked positions show the development of the Al and Ca ions in the pore solution over time in this environment. (**b**) A logarithmic presentation of (**a**), giving insight in the effect of small scale changes at low Ca content. The circled zone marks the area where the system is heading.

For a proper modelling which is in accordance with the experimental findings even minor amounts of S (<1 mmol/L) in the pore solution have to be taken into account [28]. This need was addressed in further calculations and the actual progress of the reaction during the different stability fields can be observed in Figure 7. 

The first pore solution composition is mainly driven by an initial dissolution of the cement phases and the calcite. The composition of the first pore solution is in a region where the solubilities of the phases formed during hydration show almost equal solubility. Hence, C_2_AH_x_ (here presented as C_2_AH_7.5_), HC, MC and AH_3_ all show supersaturation and as a consequence all phases are formed during the early hydration.

However, it can be seen that the pore solution follows the solubility curve of MC during the whole hydration. The reason for this is, that the final composition of the pore solution will be in equilibrium with the thermodynamically stable phases MC and AH_3_, which is indicated as a grey circle in Figure 7b. As a consequence, the pore solution drifts in the direction of equilibrium following the solubility of MC. At around 18 h the pore solution crosses the solubility curve of C_2_AH_7.5_.

At low Al values solubilities of CAH_10_, C_2_AH_7,5_, C and HC differ significantly. Hence, following the ion content compared to its solubility curve C_2_AH_7,5_ is dissolved again. 

The presence of S showed the necessity of including all ions in the calculations, shifting the solubility curves and leading to a fitting model of the reaction, able to describe the measured phase content. 

### 4.3. Solubility Curves and the Influence of Alkalis on Pore Solution Evolution in the Commercial CA-Cement

**Figure 8:**
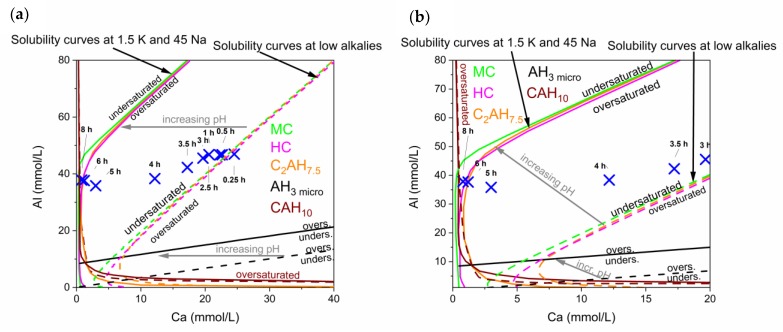
(**a**) Solubility curves in the presence of different alkali contents. Low alkalis means 0.05 mmol/L K, 0.5 mmol/L Na (dotted lines); the representative curves for high alkali with 1.5 mmol/L K and 45 mmol/L Na are shown as solid lines. Marked positions show the development of the pore solution over time in the CA-cement. (**b**) Is a close up of the development from 3 h to 8 h of hydration.

There is a noticeable increase of Na and K during hydration of the commercial CAC. Therefore, solubility curves for high and low alkali contents were plotted in the same diagram, in order to show the development of the pore solution in the commercial CAC system. The evolution of the pore solution during this reaction and the different solubility curves can be observed in Figure 8. 

It has to be considered that the reaction is never static but an ongoing process, leading to an increase of the concentration of alkalis in the pore water over time (no alkali phases can precipitate). Thus, the solubility curves shift during hydration time to lower calcium concentrations, as indicated with arrows in Figure 8. 

During the complete reaction the solution is supersaturated with respect to microcrystalline AH_3_. A low crystalline form of AH_3_ is the first phase that can be observed by QXRD slightly before 5 h. Predicted precipitation of other phases like MC, HC, C_2_AH_7,5_ and CAH_10_ can be correlated to phases observed by XRD starting from 5 h onwards.

Again initial dissolution leads to supersaturation of all phases during the first 5 h of reaction. Pore solution at times later than 5 h evolves alongside with equilibrium to the stable phases (MC, AH), in accordance to GEMS modelling shown in chapter 4.1. 

At low Ca values solubilites of C_2_AH_7,5_, MC and HC differ, as can be observed in the enlarged Figure 8b. The solubility curve of C_2_AH_7,5_ is crossed and formed C_2_AH_x_ dissolves from 6 h onwards.

**Figure 9:**
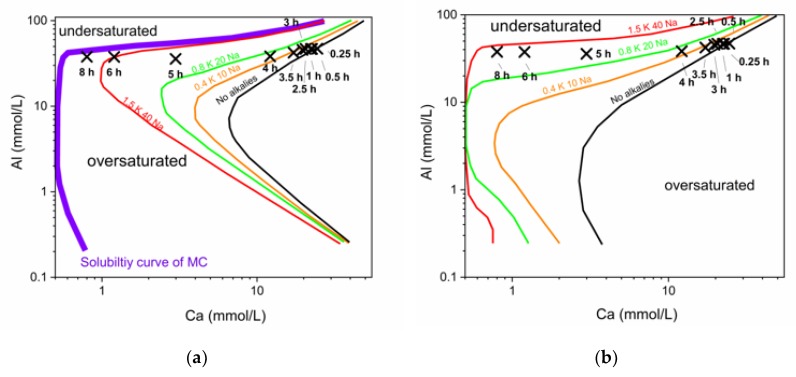
Development of solubility curves of C_2_AH_7.5_ (**a**) and monocarbonate (**b**) with changing alkali concentrations (in mmol/L). The logarithmic display gives more insight in the effect of small scale changes at low Ca content in the pore solution.

To show the immense impact of an increase of alkali content on the solubility of a hydrate phase, curves for two examples are given in Figure 9. It can be seen that there is a significant shift of the solubility curves of C_2_AH_7.5_ (Figure 9a) and MC (Figure 9b) with increasing alkalis. Hence, it can be observed why the pore solution composition of the commercial CAC + CaCO_3_ system drifts to lower Ca values. The pore solution will be in equilibrium with the thermodynamically stable phases at the end of hydration, which are MC and AH_3_ in our system. During hydration the pore solution always follows the solubility curve of the stable phase MC. Hence, the drift of the pore solution composition is in accordance with the drift of the solubility curves as indicated in Figure 9b. 

## 5. Conclusions

**Impact of ion content on the path of reaction:**

The comparison of a pure CA & calcite mix to a CA-cement & industrial calcite mix enabled insight in the influence of alkali-release into pore solution on the possible paths of reaction. A similar direction of the Ca to Al ratio was observed, although the total ratios varied significantly in pure CA and CA-cement mix. The solubility curves are shifting over time. This effect is connected to the increase of alkalis due to increasing concentration of the pore solution.

**Impact of alkalis on the solubility of hydrate phases:**

With increasing Na & K content the solubilities curves of C_2_AH_7.5_ and MC in the pore water shift. Supersaturation occurs already at a lower Ca content. Precipitation is possible at very low Ca concentrations in the end of the hydration reaction because of the increased alkali concentrations. 

**Pore solution always follows the solubility of the stable phase:**

In our system the thermodynamic stable phase is monocarbonate. During the hydration the pore solution always follows the solubility curve of this phase. This development is fueled by the ongoing precipitation of monocarbonate and further dissolution of the reactive CA, thus following an equilibrium along the solubility curve. At the end, the zone where both, monocarbonate and AH_3_ coexist must be reached. This is the case where the respective solubility curves intersect.

**Future outlook:**

The understanding of the reaction paths and resulting phase formations can be greatly enhanced by thermodynamic calculations. This knowledge might be of use for creating future experiment set ups that utilize a certain artificial ion content to influence the reaction path into a pre calculated hydration regime. 

## Figures and Tables

**Table 1:** Chemical analysis of used calcite and CA sources. (* data as published in [2]).

Oxide	Commercial Calcite * (wt%)		High Purity Calcite (wt%)		Commercial CA-cement * (wt%)		Pure CA (wt%)	
SiO_2_	0.26	±0.04	0.19	±0.04	0.39	±0.04	0.09	±0.04
TiO_2_	0.020	±0.00	0.017	±0.004	0.024	±0.004	0.018	±0.00
Al_2_O_3_	0.09	±0.05	<0.0038	-	63.67	±0.05	64.25	±0.05
Fe_2_O_3_	0.09	±0.04	0.06	±0.04	0.16	±0.04	0.05	±0.04
Mn_2_O_3_	<0.0013	-	0.001	±0.001	0.01	±0.001	0.001	±0.00
MgO	2.15	±0.02	0.233	±0.02	0.21	±0.02	<0.0034	-
CaO	51.85	±0.34	55.99	±0.34	34.27	±0.34	35.15	±0.34
Na_2_O	0.10	±0.01	0.10	±0.01	<0.014	-	<0.014	-
K_2_O	0.03	±0.02	0.02	±0.02	0.02	±0.02	0.02	±0.02
P_2_O_5_	10.73	±0.02	0.09	±0.02	0.07	±0.02	0.13	±0.02
LOI	34.63	-	43.26	-	1.13	-	0.27	-

**Table 2:** QXRD results from G-factor method (* data as published in [2]).

Phase	Commercial Calcite * (wt%)		High Purity Calcite (wt%)		Commercial Ca-Cement * (wt%)		Pure CA (wt%)	
Calcite	73.2	±0.4	100	±0.7	-	-	-	-
Dolomite	7.1	±0.1	-	-	-	-	-	-
CA	-	-	-	-	86.9	±1.9	100	±0.3
CA_2_	-	-	-	-	4.7	±0.2	-	-
Not quantified by XRD	19.8	±0.3	-	-	8.5	±2.1	-	-

**Table 3:** Compositions of the two investigated pastes in (g).

Samples	CA Source	CaCO_3_ Source	H_2_O
Pure CA+ pure CaCO_3_	70	30	100
CA-cement + commercial CaCO_3_	70	30	100

## Appendix A

**Table A1:** Settings for powder and in-situ XRD (Bruker D8 Advance, Lynx Eye detector).

Settings	Dry Samples	In-Situ XRD/Pastes
Setup	θ–2θ	θ–2θ
Goniometer radius	217 mm	217 mm
Radiation	Cu-Kα	Cu-Kα
Voltage	40 kV	40 kV
Current	40 mA	40 mA
Angular range	7–70° 2θ	6–54° 2θ
Step size	0.011° 2θ	0.0236° 2θ
Counting time/step	0.3 s	0.27 s
Divergence slit	0.3°	0.3°
Specimen rotation	No rotation	No rotation
Detector	Lynx Eye	Lynx Eye

**Table A2:** Applied crystal structures and models for Rietveld refinement.

Phase	Authors	Used Model
CA	Hoerkner and Mueller Buschbaum 1976 [29]	ICSD-260
CA_2_	Goodwin and Lindop 1970 [30]	ICSD-14270
Cc (Calcite)	Malsen et al. 1995 [31]	ICSD-80869
Gibbsite AH_3_	Saalfeld and Wedde 1974 [32]	ICSD-6162
CAH_10_	Guirado 1998 [33]	ICSD-407150
HC (Calcium Hemicarboaluminate)	Runcevski et al. 2012 [34]	ICSD-263124
MC (Calcium Monocarboaluminate)	Francois et al. 1998 [35]	ICSD-59327
C_2_AH_8_	Raab 2010 [36]	Own hkl phase model generated
Water	Bergold et al. 2013 [37]	hkl phase model adopted from Bergold et al. 2013 [23]
Kapton Film	Bergold et al. 2013 [37]	hkl phase model adopted from Bergold et al. 2013 [23]
Katoite	Lager et al. 1978 [38]	ICSD-202315

## References

[1] Manninger T., Jansen D., Neubauer J., Goetz-Neunhoeffer F. (2019). The Retarding Effect of Phosphoric Acid during CAC Hydration. Cem. Concr. Res..

[2] Manninger T., Jansen D., Neubauer J., Goetz-Neunhoeffer F. (2019). Accelerating Effect of Li_2_CO_3_ on Formation of Monocarbonate and Al-Hydroxide in a CA-Cement and Calcite Mix during Early Hydration. Cem. Concr. Res..

[3] Scrivener K.L., Capmas A. (2001). Calcium Aluminate Cements 2001. Lea’s Chemistry of Cement and Concrete.

[4] Andersson K., Allard B., Bengtsson M., Magnusson B. (1989). Chemical Composition of Cement Pore Solutions. Cem. Concr. Res..

[5] De Oliveira I.R., De Andrade T.L., Parreira R.M., Jacobovitz M., Pandolfelli V.C. (2015). Characterization of Calcium Aluminate Cement Phases When in Contact with Simulated Body Fluid. Mater. Res..

[6] Ukrainczyk N., Vrbos N., Šipušić J. (2012). Influence of Metal Chloride Salts on Calcium Aluminate Cement Hydration. Adv. Cem. Res..

[7] Currell B.R., Grzeskowlak R., Mldgley H.G., Parsonage J.R. (1987). The Acceleration and Retardation of Set High Alumina Cement by Additives. Cem. Concr. Res..

[8] Kuzel H.-J., Baier H. (1996). Hydration of Calcium Aluminate Cements in the Presence of Calcium Carbonate. Eur. J. Mineral..

[9] Midgley H.G., Midgley A. (1975). The Conversion of High Alumina Cement. Mag. Concr. Res..

[10] Neville A. (2009). History of High-Alumina Cement. Part 2: Background to Issues. Proc. Inst. Civ. Eng. Eng. Hist. Herit..

[11] Bizzozero J., Scrivener K.L. (2015). Limestone Reaction in Calcium Aluminate Cement-Calcium Sulfate Systems. Cem. Concr. Res..

[12] Luz A.P., Pandolfelli V.C. (2012). CaCO_3_ Addition Effect on the Hydration and Mechanical Strength Evolution of Calcium Aluminate Cement for Endodontic Applications. Ceram. Int..

[13] Damidot D., Stronach S., Kindness A., Atkins M., Glasser F.P. (1994). Thermodynamic Investigation of the CaO Al_2_O_3_ CaCO_3_ H_2_O Closed System at 25 °C and the Influence of Na_2_O. Cem. Concr. Res..

[14] Jansen D., Goetz-Neunhoeffer F., Stabler C., Neubauer J. (2011). A Remastered External Standard Method Applied to the Quantification of Early OPC Hydration. Cem. Concr. Res..

[15] Hertel T., Neubauer J., Goetz-Neunhoeffer F. (2016). Study of Hydration Potential and Kinetics of the Ferrite Phase in Iron-Rich CAC. Cem. Concr. Res..

[16] Lothenbach B., Kulik D.A., Matschei T., Balonis M., Baquerizo L., Dilnesa B., Miron G.D., Myers R.J. (2019). Cemdata18: A Chemical Thermodynamic Database for Hydrated Portland Cements and Alkali-Activated Materials. Cem. Concr. Res..

[17] Paul Scherrer Institut Villigen GEMS Software Main Page. http://gems.web.psi.ch.

[18] Kulik D.A., Wagner T., Dmytrieva S.V., Kosakowski G., Hingerl F.F., Chudnenko K.V., Berner U.R. (2012). GEM-Selektor Geochemical Modeling Package: Revised Algorithm and GEMS3K Numerical Kernel for Coupled Simulation Codes. Comput. Geosci..

[19] Wagner T., Kulik D.A., Hingerl F.F., Dmytrievava S.V. (2012). Gem-Selektor Geochemical Modeling Package: TSolMod Library and Data Interface for Multicomponent Phase Models. Can. Mineral..

[20] Zajac M., Skocek J., Bullerjahn F., Lothenbach B., Scrivener K., Ben Haha M. (2019). Early Hydration of Ye’elimite: Insights from Thermodynamic Modelling. Cem. Concr. Res..

[21] Lothenbach B., Zajac M. (2019). Application of Thermodynamic Modelling to Hydrated Cements. Cem. Concr. Res..

[22] Jansen D., Wolf J.J., Fobbe N. (2020). The Hydration of Nearly Pure Ye’elimite with a Sulfate Carrier in a Stoichiometric Ettringite Binder System. Implications for the Hydration Process Based on in-Situ XRD, 1H-TD-NMR, Pore Solution Analysis, and Thermodynamic Modeling. Cem. Concr. Res..

[23] Parkhurst D., Appelo C. (2013). Description of Input and Examples for PHREEQC Version 3—a Computer Program for Speciation, Batch-Reaction, One-Dimensional Transport, and Inverse Geochemical Calculations.

[24] Schreiner J., Jansen D., Ectors D., Goetz-Neunhoeffer F., Neubauer J., Volkmann S. (2018). New Analytical Possibilities for Monitoring the Phase Development during the Production of Autoclaved Aerated Concrete. Cem. Concr. Res..

[25] Klaus S.R., Neubauer J., Goetz-Neunhoeffer F. (2015). How to Increase the Hydration Degree of CA—The Influence of CA Particle Fineness. Cem. Concr. Res..

[26] Klaus S.R., Neubauer J., Goetz-Neunhoeffer F. (2016). Influence of the Specific Surface Area of Alumina Fillers on CAC Hydration Kinetics. Adv. Cem. Res..

[27] Hueller F., Naber C., Neubauer J., Goetz-Neunhoeffer F. (2018). Impact of Initial CA Dissolution on the Hydration Mechanism of CAC. Cem. Concr. Res..

[28] Lothenbach B., Pelletier-Chaignat L., Winnefeld F. (2012). Stability in the System CaO–Al_2_O_3_–H_2_O. Cem. Concr. Res..

[29] Hörkner W., Müller-Buschbaum H. (1976). Zur Kristallstruktur von CaAl_2_O_4_. J. Inorg. Nucl. Chem..

[30] Goodwin D.W., Lindop A.J. (1970). The Crystal Structure of CaO.2Al_2_O_3_. Acta Crystallogr. Sect. B Struct. Crystallogr. Cryst. Chem..

[31] Maslen E.N., Streltsov V.A., Streltsova N.R., Ishizawa N. (1995). Electron Density and Optical Anisotropy in Rhombohedral Carbonates. III. Synchrotron X-Ray Studies of CaCO_3_, MgCO_3_ and MnCO_3_. Acta Crystallogr. Sect. B Struct. Sci..

[32] Saalfeld H., Wedde M. (1974). Refinement of the Crystal Structure of Gibbsite, Al(OH)_3_. Zeitschrift fur Krist.-New Cryst. Struct..

[33] Guirado F., Galí S., Chinchón S., Rius J. (1998). Crystal Structure Solution of Hydrated High-Alumina Cement from X-Ray Powder Diffraction Data. Angew. Chemie Int. Ed..

[34] Runčevski T., Dinnebier R.E., Magdysyuk O.V., Pöllmann H. (2012). Crystal Structures of Calcium Hemicarboaluminate and Carbonated Calcium Hemicarboaluminate from Synchrotron Powder Diffraction Data. Acta Crystallogr. Sect. B Struct. Sci..

[35] François M., Renaudin G., Evrard O. (1998). A Cementitious Compound with Composition 3CaO.Al_2_O_3_.CaCO_3_.11H_2_O. Acta Crystallogr. Sect. C Cryst. Struct. Commun..

[36] Raab B. (2013). Synthese Und Charakterisierung Nanoskaliger Hydraulisch Hochreaktiver Phasen Des Portland-Und Tonerdezements.

[37] Bergold S.T., Goetz-Neunhoeffer F., Neubauer J. (2013). Quantitative Analysis of C–S–H in Hydrating Alite Pastes by in-Situ XRD. Cem. Concr. Res..

[38] Lager G.A., Armbruster T., Faber J. (1987). Neutron and X-Ray Diffraction Study of Hydrogarnet Ca_3_Al_2_(O_4_H_4_)_3_. Am. Mineral..

